# Editorial: Understanding membrane transporters: from structure to function

**DOI:** 10.3389/fmolb.2023.1323824

**Published:** 2023-10-30

**Authors:** Andrea Pasquadibisceglie, Maria Carmela Bonaccorsi Di Patti, Daniela Valeria Miniero, Giovanni Musci, Fabio Polticelli

**Affiliations:** ^1^ Science for Life Laboratory, Department of Applied Physics, KTH Royal Institute of Technology, Solna, Sweden; ^2^ Department of Biochemical Sciences “A. Rossi Fanelli”, Sapienza University of Rome, Rome, Italy; ^3^ Department of Biosciences, Biotechnologies and Environment, University “Aldo Moro” of Bari, Bari, Italy; ^4^ Department of Biosciences and Territory, University of Molise, Pesche, Italy; ^5^ Department of Sciences, University Roma Tre, Rome, Italy; ^6^ National Institute of Nuclear Physics, Roma Tre Section, Rome, Italy

**Keywords:** membrane transporters, protein structure, protein function, molecular modeling, molecular dynamics

## Understanding membrane transporters: from structure to function

Membrane transporters are critical to the passage of molecules across biological membranes and compartments, including the extracellular and intracellular environments. For this reason, these proteins play a crucial role in a plethora of patho-physiological processes in all organisms and as such are of great interest from the biomedical and biotechnological viewpoints. They include primary and secondary active and passive transporters which exert their function through conformational changes induced by ligand binding or other mechanisms including, among others, allosteric gating, transmembrane potential variation, and ATP hydrolysis.

This Research Topic collects scientific contributions concerning the study of membrane transporters, with a specific focus on the characterization of the relationships between their structure, dynamics, and mechanism of action through a combination of experimental and computational techniques. They also include studies on the interactions between transporters and membrane lipids and how these affect the transport mechanism.

Hang and coworkers carried out a homology modeling and molecular dynamics study of Sx_c_^−^, a Na^+^-independent amino acid antiporter belonging to the Solute Carrier Family 7 of transporters (SLC7) that regulates the cellular uptake of cystine in exchange for glutamate across the plasma membrane. Sx_c_^−^ is considered a possible target for the treatment of cancer and neurodegenerative diseases as it is overexpressed in several cancer types and plays a role in neurodegenerative diseases. The authors present an effort to construct high-quality structural models of this transporter in different conformations along the transport pathway, uncovering for the first time the structure of both N- and C- terminal domains, using multi-template homology modeling followed by refinement through molecular dynamics simulations. Validation of the approach has been successfully obtained through comparison with the Cryo-EM structures of Sx_c_^−^ in the inward-open conformation, which, however, lacks structural information on the N- and C-termini exerting a regulatory role. Therefore, this is a good example highlighting the complementarity between experimental and computational techniques in providing a complete picture of the conformational transitions involved in the translocation of the ligands by membrane transporters.

Mishra and colleagues provide additional insights into the mechanism underlying the different functions of two insertases, YidC1 and YidC2, expressed in Gram-positive bacteria. These proteins belong to the YidC/Oxa1/Alb3 family, which is conserved in all three domains of life. To date, the functional differences between the two paralogs have been attributed to their cytoplasmic domains. A combination of molecular modeling, coarse-grained molecular dynamics (CGMD) simulations, molecular genetics, and phenotypic characterization allowed the authors to investigate the coordinated cardiolipin-YidC2 activity in response to acid and envelope stress. In detail, cardiolipin (CL) molecules are proposed to establish different specific interactions with YidC1 and YidC2 dimers. Mutations of two specific orthologous residues affect both the paralog-specific function and the relative CL-interaction profiles. These new results enrich the current vision of the membrane protein insertion machinery of Gram-positive organisms, highlighting how the membrane composition could affect this process.

The article by Boakes and colleagues deals with equilibrium nucleoside transporters (ENTs), a major family of nucleoside transporters representing important pharmacological targets. The available crystal structure has several unsolved portions, but it revealed a peculiar outward-facing conformation similar to those of the major facilitator superfamily (MFS) members. However, unlike MFS transporters, ENTs have 11 rather than 12 transmembrane (TM) helices and lack the so-called A motif, which is essential for transport activity in many MFS transporters. Through stability and inhibitor assays, the authors investigate the human ENT isoform 1 (hENT1) variants, both unbound (apo-hENT) and bound to the NBMPR inhibitor. The introduction of a new protocol for bacmid DNA extraction is noteworthy. Variants on ICL6, one of the intracellular loops, and on the adjacent TM7 have been found to stabilize both apo- and NBMPR-bound-hENT1. These variants are proposed to contribute to the intracellular gating and to the interactions with the lipid bilayer. The evidence for the ICL6 importance, with the help of new structural data, could better explain the inhibitor and substrate sensitivity of hENT1.

The article by Luo and coworkers reports the cryo-EM structure of Dispatched (Disp), a protein involved in the transport of hedgehog proteins, solved at 6.5 Å resolution. Disp belongs to the resistance-nodulation-division (RND) family, characterized by a pseudosymmetric arrangement of two-halves composed of 6 TMs and a large extracellular domain each. This article is an excellent example of the strategy generally employed for structural studies of membrane proteins, which are mostly difficult to crystallize. The authors first attempt to produce the mouse Disp; then, they turn to homologs from simpler organisms to obtain higher amounts of high-quality recombinant protein. In this case, the water bear Disp proved to be the best choice, and further optimization by deleting predicted flexible regions led to monodisperse particles that could be analyzed by cryo-EM. Structural features of water bear Disp appear to be slightly different from those reported for *drosophila* and human Disp at the TM regions, suggesting that the former might have been captured in a different conformation. This result is particularly intriguing, with the caveat of the low resolution, because obtaining snapshots of membrane transporters in different conformations is crucial for understanding their mechanism of action.

The contributions collected in this Research Topic are good examples of the research focused on the structure and function of membrane proteins, specifically membrane transporters, where the environment (*e.g.*, the lipid bilayer membrane) plays a critical role and has to be taken into account. This Research Topic also offers a view of the challenges involved in the study of these biological systems. Challenges that, these days, can be addressed by combining experimental and computational methods, with the latter being easily exploited to test and suggest new hypotheses.

